# Impact of air pollution on mortality: Geo-epidemiological study in French-speaking Africa

**DOI:** 10.1016/j.heliyon.2024.e39473

**Published:** 2024-10-16

**Authors:** Laurie Capitanio, Sylviane Ratte, Sylvain Gautier, Loic Josseran

**Affiliations:** a Département Universitaire Santé Publique, Observation, Territoires, UFR Simone Veil-santé, University of Versailles Saint-Quentin, 2 Av. de la Source de la Bièvre, 78180, Montigny-le-Bretonneux, France; b Vital Strategies – European Office, 9 rue Charlot, 75003, Paris, France; c CESP U1018 Inserm, Équipe "soins Primaires, Prévention", 16 av, Paul Vaillant Couturier, 94807, Villejuif, France; d Département Hospitalier D'épidémiologie Et De Santé Publique, Hôpital Raymond Poincaré, Ap-Hp, 104 Bd Raymond Poincaré, 92380, Garches, France

**Keywords:** Air pollution, PM_2.5_, Cardiorespiratory diseases, Spatial interpolation

## Abstract

According to the World Health Organization, air pollution is responsible for 90 % of deaths in Africa. However, limited data on exposure to air pollution is available, and studies are rare, particularly in French-speaking Africa. This study aims to investigate the impact of air pollution on mortality in 12 French-speaking African countries (Algeria, Burkina Faso, Burundi, Cameroon, Guinea, Ivory Coast, Madagascar, Mali, Morocco, Democratic Republic of Congo, Senegal, Tunisia). Using data from the Institute for Health Metrics and Evaluation (IHME), annual concentrations of the PM_2.5_ pollutant from different cities were integrated into a spatial interpolation model (IDW) at the scale of each country. The interpolation was validated using cross-validation models. For each of the considered cardiorespiratory diseases (LRI, stroke, COPD, IHD), the attributable mortality fraction was estimated using literature data and population exposure calculated using demographic data from each country. Large variations in ambient PM_2.5_ concentration between the 12 countries are observed, with concentrations ranging from 1.76 μg/m^3^ in Morocco to 64.99 μg/m^3^ in Cameroon. Concentrations are higher in West Africa than in Central or North Africa. In 2019, 136 457 deaths attributable to air pollution were recorded in the 12 countries: 40.8 % from ischemic heart disease, 38.5 % from stroke, 13.2 % from lower respiratory infections and 7.5 % from chronic obstructive pulmonary disease. Our model allowed us to obtain a spatial distribution and the number of deaths related to air pollution. However, the estimation of the health impact from air pollution could be improved by more systematic and comprehensive local exposure assessments from a robust air quality monitoring system.

## Highlights

• Study estimates over 136 000 deaths from air pollution in 2019 across 12 countries.
• GIS methods reveal spatial PM2.5 distribution in 12 French-speaking African countries.
• West Africa has higher PM2.5 annual concentrations than North Africa.
• Study emphasizes need for more air quality monitoring and mortality data in the region.

## Abbreviations:

COPD: chronic obstructive pulmonary disease
GIS: geographic information system
IDW: inverse distance weighted interpolation method
IHME: Institute for Health Metrics and Evaluation
IHD: ischemic heart disease
LRI: lower respiratory infection
NCD: non-communicable disease
PAF: population attributable fraction
PM_2.5_: particulate matter lower than 2.5 μg/m^3^
RMSE: root of the mean square of error
RR: relative risk

## 1 Introduction

Air pollution is one of the most worrying environmental problems of our time. On a global scale, it significantly affects human health, the ecosystem, and the climate. Epidemiological studies have shown that exposure to ambient air pollution leads to adverse health effects, including increases in mortality and morbidity from cardiovascular and respiratory diseases [1]. Mortality attributed to ambient air pollution is identified as an indicator of the sustainable development goals (SDGs) [2]. There are different air pollutants, and the sources of emissions differ from region to region and can be both anthropogenic and natural. Fine particulates (PM_2.5_), the most health impactful of pollutants, are a major risk factor for health, particularly for non-communicable diseases (NCDs), affecting every organ in the body. Both acute and chronic exposure to these particles increases the risk of respiratory and cardiovascular diseases, including lung cancer and stroke [3,4]. The Global Burden of Disease study estimates that over 4 million deaths are attributable to ambient air pollution [5].

About 1.1 million deaths are linked to air pollution in Africa each year [6]. Africa is a rapidly growing economic and demographic continent that faces major air pollution challenges [7,8]. Rapid urbanization, increasing industrialization and a growing number of petroleum-powered vehicles have contributed to a deterioration of air quality in many African regions [7,9]. In addition, traditional practices such as biomass burning for cooking and domestic heating are still commonly used in many households, contributing significantly to high levels of indoor and ambient PM_2.5_.

Our study focuses on French-speaking Africa, a region that shared colonial history and unique socio-political dynamics [10]. The common language across these countries creates a unified framework for public administration and health systems. This linguistic unity creates a cohesive framework through which health issues are identified, studied, and more easily addressed. These countries are part of different economic groups that promote development collaborations, such as Economic Community of West African States (15 countries, nine of which are francophone), the Economic Community of Central African States (11 countries, nine francophone), and the Southern African Development Community (16 countries, four francophone) [11]. That allow them to benefit from strong financial and diplomatic networks, but also, the sharing of the same official language allows them to have enabling coordinated responses to common public health challenges.

Despite high PM_2.5_ concentrations in some African cities, particularly in francophone Africa, only a few studies describing this pollution are available. Indeed, most research on air pollution in Africa has focused on English-speaking countries, leaving a gap in the knowledge available for French-speaking countries [12]. The lack of sufficient air quality data means that many national and local governments have limited knowledge about emission sources, concentrations, and trends. It also means that the effectiveness of predictive models used by some governments may be limited, as they require ground-level measurements to be accurate [12].

The originality of this research lies in its sub-national focus, which has not been previously examined in this region. The mainobjective of this study is to carry out a geo-epidemiological analysis of the impact of PM_2.5_ air pollution in francophone African countries. More precisely, based on data on concentrations of this pollutant and its spatial distribution, the mortality attributable to PM_2.5_ air pollution is estimated. This approach seeks to provide essential data that will help policy-makers understand the wider effects of air pollution, thus providing a basis for informed decision-making in French-speaking Africa.

## 2 Material and methods

In our study, we used a geo-epidemic method, using a geographic information system (GIS) to measure the impact of pollution. We had to quantify the pollution, population, and the impact through a model. The modeling method using GIS was computed in QGIS 3.26.3 [1,13,14].

### 2.1 Study area

French-speaking Africa refers to all African states that share French language. In total, 21 African states have French as an official language. We focused on 12 French-speaking countries (Fig. 2): 3 in North Africa: Algeria, Morocco, Tunisia; 5 in West Africa: Burkina Faso, Guinea, Ivory Coast, Mali, Senegal; 4 in Central Africa: Burundi, Cameroon, Democratic Republic of Congo and Madagascar.

**Fig. 1:**
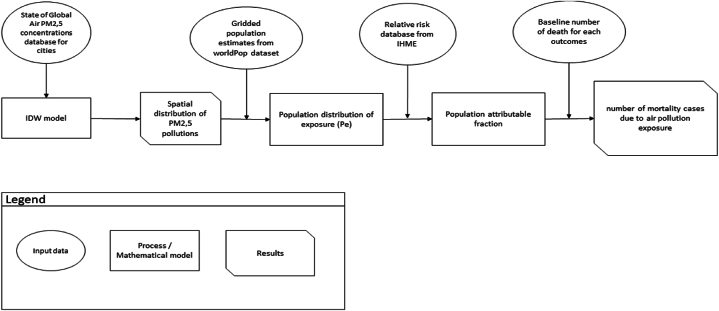
Diagram of the method used The general framework is described on Fig. 1.

**Fig. 2:**
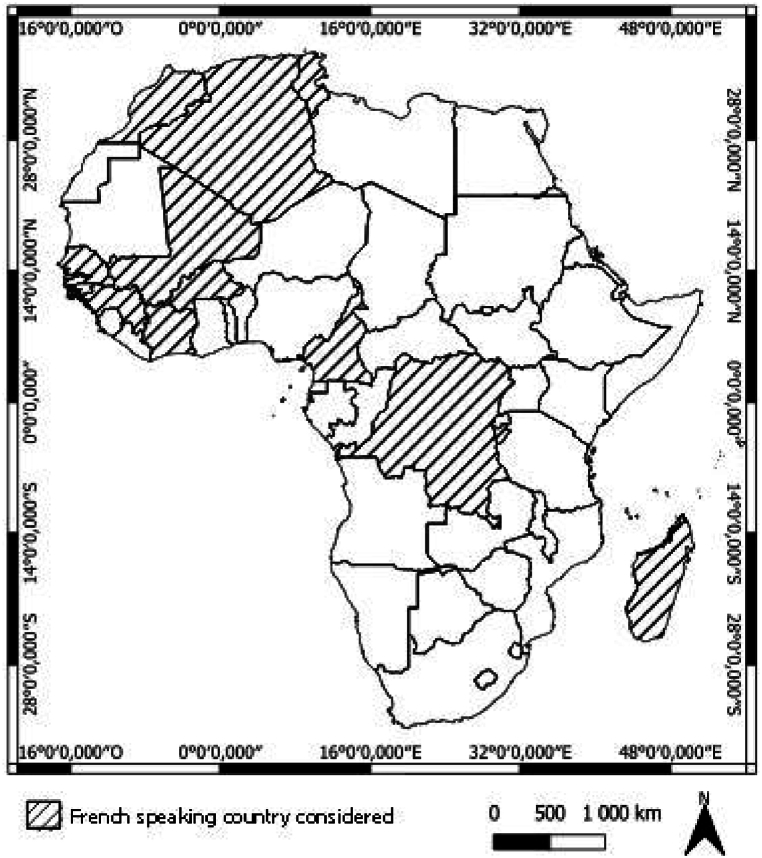
**Map of Africa.** Shaded countries are the French-speaking country we considered.

The region's countries exhibit significant demographic and economic diversity. With a combined population of over 325 million, urbanization rates vary widely, from 13 % in Burundi to 73.3 % in Algeria [15]. Higher urbanization correlates with increased exposure to air pollution due to concentrated industrial activities and traffic, which can exacerbate health problems [16,17].

Economically, there is a pronounced gap in GDP per capita, with wealthier nations in North Africa (Algeria, Tunisia and Morocco) with higher GDPs per capita above $3,000, while poorer countries like Burundi and the Democratic Republic of Congo struggle with figures below $600 [15]. This economic disparity impacts health expenditure, with wealthier countries able to invest more in pollution control and healthcare infrastructure. For instance, Algeria, with the highest current health expenditure at $236 per capita ($US), is better positioned to address pollution-related health issues compared to countries like Madagascar, which spends only $15.89 per capita [15]. Consequently, life expectancy ranges from 59 years in Côte d'Ivoire to 76 years in Tunisia, (life expectancy) reflecting the broader impact of economic resources and healthcare access on managing the health effects of air pollution [18]. Insufficient investment in the health sector or in actions to tackle the environmental and social determinants of health is a serious obstacle to improving health outcomes in Africa.

Also, these countries have several land uses as cities, villages, agricultural, and industrial regions. These regions are exposed to different climates: equatorial (dense forest), humid tropical (savannah), dry tropical (steep), arid (hot desert) and Mediterranean (dry forest), each with different attributes and impact on air pollution [19,20].

These countries have several land uses as cities, villages, agricultural, and industrial regions. These regions are exposed to different climates: equatorial (dense forest), humid tropical (savannah), dry tropical (steep), arid (hot desert) and Mediterranean (dry forest), each with different attributes and impact on air pollution [19,20].

### 2.2 Spatial air pollution distribution

Using population-weighted ambient particulate matter pollution data of the *Explore the Data | State of Global Air* webstite [21], at cities level, we estimate the spatial distribution of PM_2.5_ in each country in 2019.

We assumed that for each country, data at city level represent a monitoring station, that we geolocated. In fact, to create a comprehensive map of PM_2.5_ concentration disparities, we required geographic coordinates along with concentration data. Since GPS coordinates for specific monitoring stations were not available, we used city coordinates as proxies. To interpolate the distribution of air pollution in the country, we had to select country with at least 4 monitoring stations. This threshold was established to ensure sufficient geographic coverage and data robustness. We selected four monitoring stations per country based on their geolocation to ensure a more accurate and representative model. This approach was taken to avoid a decline in model quality that can occur with an irregular sample distribution. Countries selected were Algeria, Burkina Faso, Burundi, Cameroon, Guinea, Ivory Coast, Madagascar, Mali, Morocco, Republic democratic of the Congo, Tunisia, and Senegal. Overall, we identified 233 monitoring stations for the 12 countries. The interpolation was done using an inverse distance weighted interpolation method (IDW). This method is both simple and effective and, assumes that geographically closer points have more similar values, which aligns well with how pollutants typically disperse in the environment [1,22,23]. This interpolation estimates the value of *Z* at a point *x0* using the value of a given number of observation points *xi*, weighted by the inverse function of the distance between the unknown and the observation point, where *λi* represents the weight function assigned to each observation point *xi* and *Z(xi)* is the measured value at *xi.*

eq1 $$\hat{Z}\left(x_{0}\right)=\sum_{i=1}^{n}\lambda_{i}Z\left(x_{i}\right)\;\text{with}\;\lambda_{i}=d_{i}^{-p}/\;\sum_{i=1}^{n}d_{i}^{-p}$$

To verify and calibrate the quality of the model, we did a cross validation evaluation: comparison of predicted and observed value. We selected randomly 1 monitoring station per province and interpolated the values to neighbouring stations. We then assessed the validity of the interpolation using the square root of the mean for the squared prediction errors (RMSE) and the correlation coefficient between the observed data at monitoring stations and predicted data by the model [1,14,24,24].

### 2.3 Population exposure

To estimates the population exposure to different level of PM_2.5_, we acquired population data from *WorldPop|* *Population Counts* *website* [25]. The dataset estimates total number of people per grid-cell at a resolution of 30 arc (approximately 1 km at the equator). The population exposure is defined as the sum of the number of people per grid (all ages and both sex) in cities exposed to different level of PM_2.5_ divided by the total population of the country. The model assumed that in each grid-cell, the population was exposed to the same PM_2.5_ concentrations level.

### 2.4 Population attributable fraction and mortality

Air pollution causes health problems such as stroke, ischemic heart disease, both chronic and acute respiratory diseases cancer. To assess the public health impact of exposures in populations, we estimated the population attributable fraction (PAF). It is defined as the fraction of all deaths of a particular disease in a population that is attributable to a specific exposure [1].

eq2 $$PAF=\sum_{i=1}^{n}{Pe}_{i}\left({RR}_{i}-1\right)/\sum_{i=1}^{n}{Pe}_{i}\left({RR}_{i}-1\right)+1$$

*Pei* = proportion estimates of the population in exposure category i *RRi* = Relative risk in exposure category ‘i’

The exposed population may be divided into multiple categories (*n*) based on the level of concentration, each with their own relative risk (*RR*). We used RR values for ambient air pollution exposure, estimated by Murray *et al*, [26] as function of exposure based on published systematic reviews, 81 systematic reviews carried out for GBD 2019 and meta-regressions. The data provide an age- and sex-specific RR for each outcome of the integrated particle exposure response curve. Thus, for a population aged over 25, we had different RR values for each PM exposure concentration and for each outcome. The RR values are available on the Institute for Health Metrics and Evaluation website [27]. We estimated the PAF for each outcome: ischemic heart disease, stroke, lower respiratory diseases, and the chronic obstructive pulmonary disease, for a population in each country of both sex and age above 25 years old.

Finally, to estimate the expected number of mortality cases due to ambient air pollution exposure *E*, we applied PAF to the number of mortalities.

eq3 E=PAF∗N

The baseline number of death for each outcome (*N*) was obtain from the IHME. From that number, we were able to attribute the percent of mortality due to ambient and household air pollution.

## 3 Results

### 3.1 Spatial distribution

The spatial distribution of air pollution was interpolated with the IDW model. Table 1 shows the statistics of the model for each country. Globally, the root mean square error is close to 0, which means that the cross-validation values are close to the measured values. Coefficients of correlation between the model predictions and the measured values at the monitoring stations were calculated. The correlation coefficients are significant for all countries, except for Burundi (p > 0.05). We obtained the minimum, maximum, mean concentration for each country with the 95 % CI.

**Table 1:** Statistics of the air quality data and spatial interpolation model of pollutant PM_2.5_.

Statistics measured	Concentrations mean (μg/m^3^)	Min (μg/m^3^)	Max (μg/m^3^)	95 % CI (μg/m^3^)	RMSE	Correlation
Algeria	17.58	3.06	41.89	[16.29, 18.87]	0.69431	0.98^a^
Burkina Faso	43.23	39.5	46.5	[39.24, 47.22]	0.023	0.99^a^
Burundi	21.27	19.3	22.3	[19.95, 22.95]	0.119	1
Cameroon	47.24	37.4	65	[37.64, 56.83]	0.118	0.99^a^
Guinea	36.75	31.7	44.2	[33.24, 40.25]	0.169	0.99^a^
Ivory Coast	44.59	30.3	50.4	[41.90, 47.28]	0.483	0.99^a^
Madagascar	4.6	3.9	5.4	[3.94, 5.25]	0.0028	0.99^a^
Mali	45.13	39.2	54.5	[42.82, 47.45]	0.058	0.99^a^
Morocco	12.66	1.7	22.4	[11.33, 13.99]	0.748	0.98^a^
Republic Democratic of the Congo	27.58	13.4	41.2	[23.95, 31.20]	0.183	0.99^a^
Tunisia	14.3	4.3	22.9	[11.69, 16.91]	0.77	0.99^a^
Senegal	34.72	9.1	47.2	[24.60, 44.85]	0.82	0.99^a^

RMSE: Root-mean-square-error.

a Correlation is significant at the 0.05 level.

**Fig. 3:**
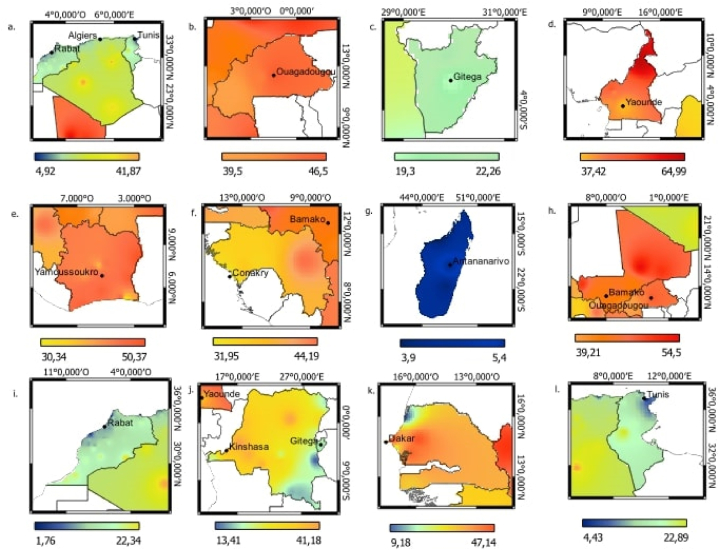
**Map of PM**_**2.5**_**concentration distribution (in μg/m**^**3**^**) in 12 Francophone African countries** a.Algeria, b.Burkina Faso, c.Burundi, d.Cameroon, e.Ivory Cost, f.Guinea, g.Madagascar, h.Mali, i.Morocco, j.Democratique Republic of the Congo, k.Senegal, l.Tunisia.

Fig. 3 visualizes the geographical distribution of annual mean estimate for exposure to PM_2.5_ air pollutants in 2019. The exposure estimated for PM_2.5_ concentrations in all French speaking countries appeared in a range from 1.76 μgm-3 to 64.99 μgm-3. The average annual PM_2.5_ concentrations in North Africa are around 20 μg/m³. In contrast, the average annual PM_2.5_ concentration in West African countries is approximately 40 μg/m³. Republic Democratic of the Congo is exposed to an average of 27.58 μg/m^3^ and the isle of Madagascar: 4.6 μg/m^3^. We can notice that West Africa have higher annual concentration than in North Africa. Country-level comparisons reveal wide disparities in PM_2.5_ exposure across the continent.

### 3.2 Population exposition

For each country, based on the spatial distribution of PM_2.5_ concentration and population per city, we determined the percentage of population exposed to different concentration levels (supplementary material) For some countries, such as Algeria, Mali, Morocco, Republic Democratic of the Congo, Tunisia and Senegal, there is a difference between the minimum and maximum concentrations allowing us to obtain different concentration levels. For other countries, such as Burundi, Burkina Faso, Guinea, Ivory Coast and Madagascar, we only have 2 different concentrations.

### 3.3 Population attributable fraction and mortality

The population attributable fraction is calculated for ischemic heart disease, stroke, chronic obstructive pulmonary disease, and lower respiratory disease, in a population that is attributable to a specific exposure: different level of PM_2.5_ concentrations. From the relative risks and the exposure of the population (older than 25yo) in the different countries, PAFs were obtained for each outcome (Table 2). In Algeria, we found that 21 %, 26 %, 17 % and 13 % of death from respectively of IHD, stroke, COPD and LRI are attributable to air pollution.

**Table 2:** Population attributable fraction (PAF) based on level of air pollution concentration and associated mortality (rounded up to the next number).

Country	Health outcome	PAF	Mortality total caused by air pollution	Mortality due to Ambient Air Pollution	Mortality due to Household Air Pollution
**ALGERIA**	Ischemic Heart Disease	0.21	12463	12089	374
	Stroke	0.26	6755	6552	203
	Chronic Obstructive Pulmonary Disease	0.17	893	866	27
	Lower Respiratory Disease	0.13	672	652	20
**BURKINA FASO**	Ischemic Heart Disease	0.29	2785	348	2437
	Stroke	0.45	3210	401	2809
	Chronic Obstructive Pulmonary Disease	0.36	487	61	426
	Lower Respiratory Disease	0.26	1854	232	1623
**BURUNDI**	Ischemic Heart Disease	0.29	1095	109	986
	Stroke	0.45	1571	157	1414
	Chronic Obstructive Pulmonary Disease	0.36	358	36	322
	Lower Respiratory Disease	0.26	636	64	572
**CAMEROON**	Ischemic Heart Disease	0.33	3479	1601	1878
	Stroke	0.47	4982	2292	2690
	Chronic Obstructive Pulmonary Disease	0.39	886	408	478
	Lower Respiratory Disease	0.27	2324	1069	1255
**IVORY COAST**	Ischemic Heart Disease	0.29	2836	851	1985
	Stroke	0.45	3783	1135	2648
	Chronic Obstructive Pulmonary Disease	0.36	696	209	487
	Lower Respiratory Disease	0.26	1923	577	1346
**GUINEA**	Ischemic Heart Disease	0.29	1714	274	1440
	Stroke	0.45	2552	408	2144
	Chronic Obstructive Pulmonary Disease	0.36	526	84	442
	Lower Respiratory Disease	0.26	1471	235	1236
**MADAGASCAR**	Ischemic Heart Disease	0.09	1043	104	934
	Stroke	0.12	1921	192	1729
	Chronic Obstructive Pulmonary Disease	0.07	230	23	207
	Lower Respiratory Disease	0.05	392	39	353
**MALI**	Ischemic Heart Disease	0.30	2286	288	1998
	Stroke	0.46	3398	427	2971
	Chronic Obstructive Pulmonary Disease	0.36	900	113	787
	Lower Respiratory Disease	0.26	799	101	698
**MOROCCO**	Ischemic Heart Disease	0.18	13226	12432	794
	Stroke	0.23	6721	6318	403
	Chronic Obstructive Pulmonary Disease	0.14	875	822	53
	Lower Respiratory Disease	0.11	588	553	35
**RDC**	Ischemic Heart Disease	0.27	8685	174	8511
	Stroke	0.39	12863	257	12606
	Chronic Obstructive Pulmonary Disease	0.29	3517	70	3447
	Lower Respiratory Disease	0.21	6057	121	5936
**TUNISIA**	Ischemic Heart Disease	0.19	4083	4071	12
	Stroke	0.24	2096	2090	6
	Chronic Obstructive Pulmonary Disease	0.15	283	282	1
	Lower Respiratory Disease	0.12	205	204	1
**SENEGAL**	Ischemic Heart Disease	0.29	2076	581	1495
	Stroke	0.45	2643	740	1903
	Chronic Obstructive Pulmonary Disease	0.35	554	155	399
	Lower Respiratory Disease	0.25	1045	293	752

For each outcome, mortality was calculated from eq (3). A total of 136 457 deaths were calculated due to air pollution in 2019. Specifically, 61 584 deaths from ambient air pollution (AAP) and 78 873 deaths from household air pollution (HAP). These deaths (AAP and HAP) were calculated from fractions of the total number of deaths (all causes) due to air pollution and the number of deaths due to ambient and indoor pollution.

## 4 Discussion

In this geo-epidemic study of atmospheric pollutant PM_2.5_ in 12 French-speaking African countries, attributable mortality associated with cardiovascular and respiratory diseases was estimated around 150 000 individuals over the age of 25yo in 2019. A GIS method was used to obtain the spatial distribution of the pollutant within the countries considered, as well as the different levels of exposure of the populations.

Our estimates of all-cause (IHD, Stroke, COPD, LRI) mortality attributable to PM_2.5_ was 6.6 % of total death in the 12 francophone countries studied. This proportion was not much different in terms of proportion compared to the *VizHub - GBD Compare*. In Africa in 2019, over 1.1 million people died prematurely from air pollution related disease [29]. According to this report, air pollution is the second leading risk factor for premature deaths after malnutrition, placing it well above unsafe water, sanitation, and hygiene, which ranked fourth largest risk factor for deaths.

The 4 diseases studied are the main causes of death due to exposure to fine particles, for all sexes worldwide in 2019 [28]. The estimated number of deaths for IHD, Stroke, COPD and LRI are 55 771, 52 495, 10 205 and 17 966 respectively. All these results are similar to those estimated by the GBD. Only our estimated number of deaths for LRIs is lower than the number of deaths estimated by the GBD, which is on average 830 274 (57,0194-115 760). This is because our calculations consider a population aged over 25. However, for this disease, it is known that it mainly affects children under the age of 5 [30,31], which explains the difference in our results.

Disparities in the number of deaths attributable to air pollution and PM_2.5_ concentration can be observed between countries. They may be linked to estimated PM_2.5_ concentrations that are well above the WHO air quality guidelines (5μg/m3) [2]. Abdolahnejad et al. [32], in their study, assess the health impact of PM_2.5_ and PM10 particles on the population of Isfahan, Iran. Their results reveal that pollution levels are well above WHO standards, contributing significantly to cardiovascular and respiratory mortality. Socio-economic conditions, healthcare availability, pollutant exposure levels and demographic differences may affect the health and vulnerability of populations to air pollution are other factors responsible for these disparities [33]. In addition, improvements in healthcare and socio-economic development can lead to a reduction in mortality rates from pollution-related diseases [11,34]. Consequently, variations in pollution-related mortality rates may be partly explained by these broader developmental changes [33]. Moreover, disparities in PM_2.5_ concentration can also be observed between countries. Our results showed lower concentrations in in North African countries, than in West African countries. Yu et al. (2024) study [34] has highlighted similar patterns to those identified in our research: observed annual PM_2.5_ concentrations are higher in sub-Saharan and West Africa than in North Africa. As previously discussed, this discrepancy can be attributed to various socio-economic factors as well as, to different sources of pollution in these regions. Owili et al. showed that there are variations in mortality effects depending on the PM_2.5_ source [35]. For example, dust from the Sahara desert contributes to high PM_2.5_ concentrations in West (sub-Saharan) and North Africa [[36], [37], [38]], impacting population health [39]. West Africa has experienced strong economic growth, particularly in cities, with the construction of road infrastructures, the development of the real estate sector, and the expansion and creation of new industrial zones. The combination of all these anthropogenic activities and demographic pressure is increasing the sources of air pollution in cities [40]. Thus, pollution from anthropogenic sources, such as industry, agricultural fires, and road traffic, is often the cause of respiratory diseases [2,36,41,42]. Although biomass-based fuels remain the main cooking fuel used in many West African countries, which is a major source of pollution [43].

Despite the burden of this pollution across the African continent, there are few studies showing its impact on health in French speaking Africa [7,19,44], most of are concentrated in South Africa [44]. Each study is thus important for understanding and assessing the regional impact of air pollution. Regarding air quality measurement systems only 17 countries in Africa have air quality standards, which are an essential component of political action to reduce air pollution and improve health [29]. Although many countries lack official references and air quality monitoring infrastructures, some have functional government air quality monitoring sites [7]. Initiatives are underway, particularly by institutions aiming to assist ECOWAS countries in launching their air quality monitoring activities [9]. Our study revealed that among the 12 Francophone African countries analyzed, few have set air quality standards. These standards, issued by national or local governments, set maximum limits for air pollutant concentrations and are essential for guiding political action to reduce air pollution and improve public health. Countries outline their actions to mitigate air pollution across various sectors, such as transport, residential, waste, agriculture, and forestry, in their Nationally Determined Contributions (NDCs). The scarcity of air quality standards in French-speaking Africa underscores the necessity to strengthen regulatory and monitoring capacities in the region. For many municipalities in low- and middle-income countries, the complexity and cost of understanding and controlling air pollution have hindered the implementation and maintenance of effective clean air actions [45]. In Africa, measurements have primarily been taken in capital cities or major urban areas, with data collected over short periods, further highlighting the need for comprehensive and sustained monitoring efforts [46].

Our study has some limitations, particularly in the use of the inverse distance weighted method. One is the fact that the weighting parameters are chosen a priori, but not empirically determined (An adaptive inverse-distance weighting spatial interpolation technique. George Y. Lu). An Another limitation is that a distance-decay parameter is applied uniformly across the entire study area without considering the distribution of data. This approach assumes that the influence of a data point decreases linearly with distance, which may not accurately reflect the spatial variability of atmospheric pollutant concentrations. These limitations can lead to less precise estimates, especially in heterogeneous environments where pollutant concentrations vary in complex ways [47].

Also, indoor pollution is a factor that we have not taken into account in our study, particularly exposure to indoor air pollution. Yet indoor pollution and its impact on health can be as important, if not more so, than outdoor pollution, and is a significant contributor to outdoor concentrations. Domestic air pollution is responsible for 697 000 deaths in Africa in 2019, compared with outdoor pollution, which is responsible for 340 000 deaths [6]. This is due to the combustion of various fuels (coal, charcoal, wood, agricultural residues, animal excrement and paraffin) for heating or cooking using open fires or stoves with limited ventilation [29]. Numerous studies have shown that the populations most vulnerable to this pollution are women and children under the age of 5 [48,49]. In 2007 the WHO estimated that more than 10 000 deaths from acute lower respiratory infections in children under five in Madagascar are attributable to solid fuel [50]. A study carried out in the city of Ouagadougou in 2018 showed that women in charge of cooking meals in households suffer from various symptoms linked to the use of biomass [43].

## 5 Conclusions

Based on the PM_2.5_ concentration data available, our innovative geo-epidemic study has enabled us to establish the current state of air quality in French-speaking Africa and to highlight the disparities between and within countries. There is a need to consolidate and deploy air quality measurement stations and to more accurately measure local causes of mortality in order to improve our knowledge of the sources of pollution and the impact of air quality on health in French-speaking Africa. Continued industrial development and increasing urbanization point to rising pollution levels. In the absence of government intervention, this upward trend in pollution could exacerbate the impact on an expanding and ageing population, increasing the risk of respiratory and cardiovascular disease for a greater number of individuals. These disparities argue in favor of drawing up mitigation and adaptation plans at a very local level. Ultimately, the fight against air pollution must be an absolute priority for all the countries of French-speaking Africa in order to protect the health and well-being of their populations, but also to preserve the environment for future generations.

## CRediT authorship contribution statement

**Laurie Capitanio:** Writing – review & editing, Writing – original draft, Visualization, Validation, Methodology, Formal analysis, Conceptualization. **Sylviane Ratte:** Writing – review & editing, Validation. **Sylvain Gautier:** Writing – review & editing, Writing – original draft, Validation, Supervision, Methodology. **Loic Josseran:** Writing – review & editing, Writing – original draft, Validation.

## Data availability statement

Has data associated with your study been deposited into a publicly available repository? No.

Has data associated with your study been deposited into a publicly available repository? Data will be made available on request.

## Funding sources

This research did not receive any specific grant from funding agencies in the public, commercial, or not-for-profit sectors.

## Declaration of competing interest

The authors declare that they have no known competing financial interests or personal relationships that could have appeared to influence the work reported in this paper.

## Appendix A Supplementary data

The following the supplementary data to this article:

## References

[1] Pinichka C., Makka N., Sukkumnoed D., Chariyalertsak S., Inchai P., Bundhamcharoen K. (Dec. 2017). Burden of disease attributed to ambient air pollution in Thailand: a GIS-based approach. PLoS One.

[2] WHO global air quality guidelines: particulate matter (PM2.5 and PM10), ozone, nitrogen dioxide, sulfur dioxide and carbon monoxide. https://www.who.int/publications-detail-redirect/9789240034228.

[3] Hamanaka R.B., Mutlu G.M. (Nov. 2018). Particulate matter air pollution: effects on the cardiovascular system. Front. Endocrinol..

[4] Song C. (Apr. 2017). Health burden attributable to ambient PM2.5 in China. Environ. Pollut. Barking Essex 1987.

[5] Vos T. (Oct. 2020). Global burden of 369 diseases and injuries in 204 countries and territories, 1990–2019: a systematic analysis for the Global Burden of Disease Study 2019. Lancet.

[6] Fisher S. (Oct. 2021). Air pollution and development in Africa: impacts on health, the economy, and human capital. Lancet Planet. Health.

[7] Mir Alvarez C., Hourcade R., Lefebvre B., Pilot E. (Jan. 2020). A scoping review on air quality monitoring, policy and health in West African cities. Int. J. Environ. Res. Publ. Health.

[8] C40, ‘Air Quality’, C40 Cities. https://www.c40.org/what-we-do/scaling-up-climate-action/air-quality/ (accessed 27 June 2023).

[9] Amegah A.K., Agyei-Mensah S. (Jan. 2017). Urban air pollution in sub-saharan Africa: time for action. Environ. Pollut..

[10] Vine V.T.L. (2004).

[11] Bcheraoui C.E. (Mar. 2020). Burden of disease in francophone Africa, 1990–2017: a systematic analysis for the global burden of disease study 2017. Lancet Glob. Health.

[12] Agbo K.E., Walgraeve C., Eze J.I., Ugwoke P.E., Ukoha P.O., Van Langenhove H. (Feb. 2021). A review on ambient and indoor air pollution status in Africa. Atmospheric Pollut. Res..

[13] Zhang C., Luo L., Xu W., Ledwith V. (Jul. 2008). Use of local Moran's I and GIS to identify pollution hotspots of Pb in urban soils of Galway, Ireland. Sci. Total Environ..

[14] Moradi Dashtpagerdi M., Sadatinejad S.J., Zare Bidaki R., Khorsandi E. (Mar. 2014). Evaluation of air pollution trend using GIS and RS applications in South west of Iran. J. Indian Soc. Remote Sens..

[15] World Bank Open Data (2019). World development indicators.

[16] Hart R., Liang L., Dong P. (Jan. 2020). Monitoring, mapping, and modeling spatial–temporal patterns of PM2.5 for improved understanding of air pollution dynamics using portable sensing technologies. Int. J. Environ. Res. Publ. Health.

[17] Liang L., Gong P. (Oct. 2020). Urban and air pollution: a multi-city study of long-term effects of urban landscape patterns on air quality trends. Sci. Rep..

[18] Maiga D.D., Eaton J. (Aug. 2014). A survey of the mental healthcare systems in five Francophone countries in West Africa: Bénin, Burkina Faso, Côte d'Ivoire, Niger and Togo. Int. Psychiatry.

[19] Imane S. (Jun. 2022). A review on climate, air pollution, and health in North Africa. Curr. Environ. Health Rep.

[20] Demir M., Dindaroğlu T., Yılmaz S. (Mar. 2014). Effects of forest areas on air quality; Aras Basin and its environment. J. Environ. Health Sci. Eng..

[21] ‘Explore the Data | State of Global Air’. https://www.stateofglobalair.org/data/#/health/plot, 2019 (accessed 20 March 2023).

[22] Masroor K. (Jun. 2020). Spatial modelling of PM2.5 concentrations in Tehran using Kriging and inverse distance weighting (IDW) methods. J. Air Pollut. Health.

[23] Kanakiya R.S., Singh S., Shah U. (Jul. 2015). GIS application for spatial and temporal analysis of the air pollutants in urban area. Int. J. Adv. Remote Sens. GIS 2015 ISSN 2320 - 0243.

[24] Weber D., Englund E. (May 1992). Evaluation and comparison of spatial interpolators. Math. Geol..

[25] WorldPop :: population counts https://hub.worldpop.org/project/categories?id=3. (accessed 27 June 2023).

[26] Murray C.J.L. (Oct. 2020). Global burden of 87 risk factors in 204 countries and territories, 1990–2019: a systematic analysis for the Global Burden of Disease Study 2019. Lancet.

[27] (2019). https://ghdx.healthdata.org/ihme_data.

[28] VizHub (2019). GBD compare.

[29] Health Effects Institute The state of air quality and health impacts in Africa. https://www.stateofglobalair.org/resources/africa.

[30] Odo D.B. (Jan. 2022). Ambient air pollution and acute respiratory infection in children aged under 5 years living in 35 developing countries. Environ. Int..

[31] Sarfo J.O. (May 2023). Acute lower respiratory infections among children under five in Sub-Saharan Africa: a scoping review of prevalence and risk factors. BMC Pediatr..

[32] Abdolahnejad A., Jafari N., Mohammadi A., Miri M., Hajizadeh Y., Nikoonahad A. (2017). Cardiovascular, respiratory, and total mortality ascribed to PM10 and PM2.5 exposure in Isfahan, Iran. J. Educ. Health Promot..

[33] H. E. Institute, ‘State of Global Air 2020 reports air pollution's impact on neonatal mortality’, Health Effects Institute. Accessed: March. 20, 2023. [Online]. Available: https://www.healtheffects.org/announcements/state-global-air-2020-reports-air-pollutions-impact-neonatal-mortality.

[34] Yu W. (Mar. 2024). Estimates of global mortality burden associated with short-term exposure to fine particulate matter (PM2·5). Lancet Planet. Health.

[35] Owili P.O., Lien W.-H., Muga M.A., Lin T.-H. (Apr. 2017). The associations between types of ambient PM2.5 and under-five and maternal mortality in Africa. Int. J. Environ. Res. Publ. Health.

[36] Bauer S.E., Im U., Mezuman K., Gao C.Y. (2019). Desert dust, industrialization, and agricultural fires: health impacts of outdoor air pollution in Africa. J. Geophys. Res. Atmospheres.

[37] De Longueville F., Hountondji Y., Ozer P., Henry S. (Oct. 2014). The air quality in african rural environments. Preliminary implications for health: the case of respiratory disease in the northern Benin. Water. Air. Soil Pollut..

[38] Sunnu A. ‘Etude expérimentale des flux et des caractéristiques physiques des poussières sahariennes dans les régions proches du golfe de guinée’, phdthesis, Université du Sud Toulon Var, 2006. https://theses.hal.science/tel-00136301.

[39] McElroy S., Dimitrova A., Evan A., Benmarhnia T. (Apr. 2022). Saharan dust and childhood respiratory symptoms in Benin. Int. J. Environ. Res. Publ. Health.

[40] Bahino J. (Apr. 2024). Temporal variability and regional influences of PM2.5 in the West African cities of abidjan (Côte d'Ivoire) and accra (Ghana). Environ. Sci. Atmospheres.

[41] Sylla F.K., Faye A., Diaw M., Fall M., Tal-Dia A. (Jan. 2018). Traffic air pollution and respiratory health: a cross-sectional study among bus drivers in dakar (Senegal). Open J. Epidemiol..

[42] Beelen R. (Feb. 2008). Long-term effects of traffic-related air pollution on mortality in a Dutch cohort (NLCS-AIR study). Environ. Health Perspect..

[43] Sana A., Meda N., Badoum G., Kafando B., Bouland C. (Mar. 2019). Primary cooking fuel choice and respiratory health outcomes among women in charge of household cooking in Ouagadougou, Burkina Faso: cross-sectional study. Int. J. Environ. Res. Publ. Health.

[44] Coker E., Kizito S. (Mar. 2018). A narrative review on the human health effects of ambient air pollution in sub-saharan Africa: an urgent need for health effects studies. Int. J. Environ. Res. Publ. Health.

[45] Strategies Vital (2022). Integrated use of low-cost sensors to strengthen air quality management in Indian cities. https://www.vitalstrategies.org/resources/integrated-use-of-low-cost-sensors-to-strengthen-air-quality-management-in-indian-cities/.

[46] Katoto P.D.M.C. (Jun. 2019). Ambient air pollution and health in Sub-Saharan Africa: current evidence, perspectives and a call to action. Environ. Res..

[47] Li Z., Wang K., Ma H., Wu Y. (Nov. 2018). Presented at the 2018 3rd International Conference on Communications, Information Management and Network Security (CIMNS 2018).

[48] Gordon S.B. (Oct. 2014). Respiratory risks from household air pollution in low and middle income countries. Lancet Respir. Med..

[49] Norman R., Barnes B., Mathee A., Bradshaw D. (Sep. 2007). South African Comparative Risk Assessment Collaborating Group.,Estimating the burden of disease attributable to indoor air pollution from household use of solid fuels in South Africa in 2000. S. Afr. Med. J..

[50] Dasgupta S., Martin P., Samad H.A. (Sep. 01, 2013). Addressing household air pollution: a case study in rural Madagascar. https://papers.ssrn.com/abstract=2333943.

