# Supplementary material for: Impact of air pollution on mortality: Geo-epidemiological study in French-speaking Africa
Source: Heliyon. 2024 Oct 16;10(20):e39473. doi: 10.1016/j.heliyon.2024.e39473 (PMC11533590; doi:10.1016/j.heliyon.2024.e39473)
Supplement: Multimedia component 1 [file mmc1.docx]

**
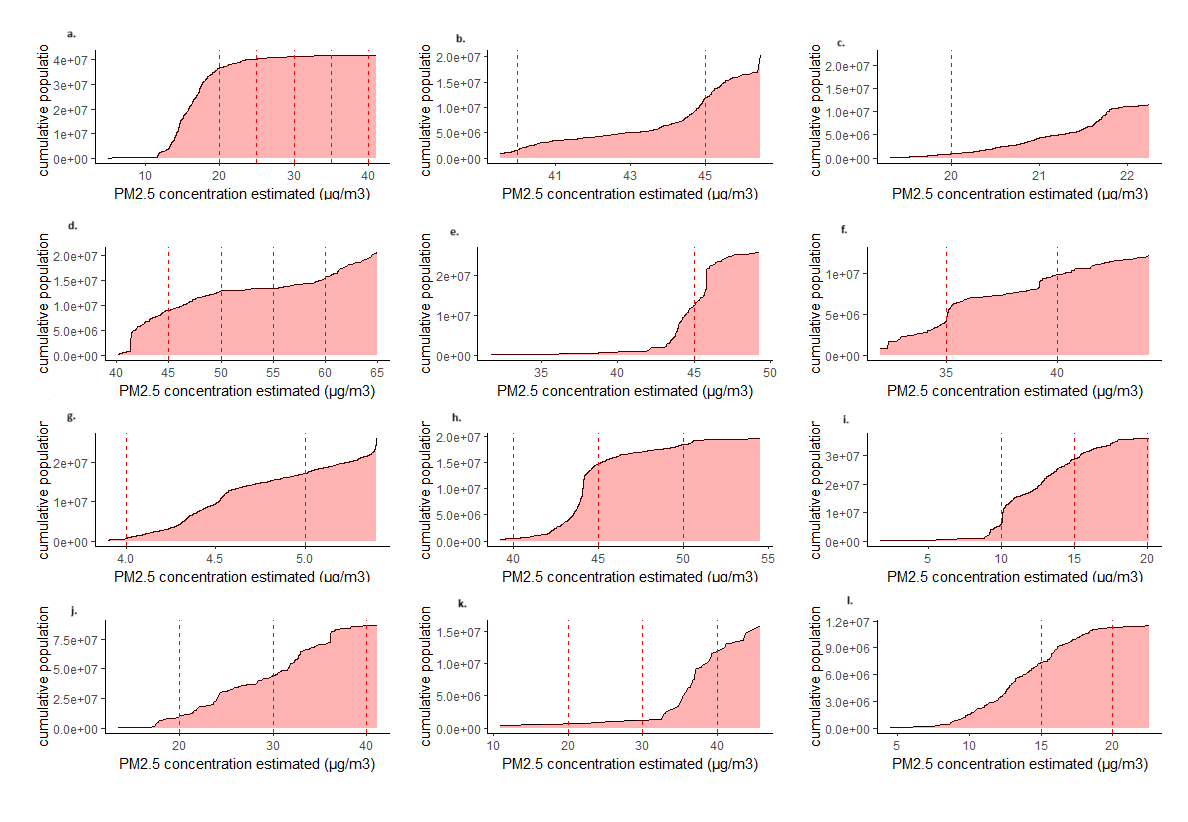
Supplementary material**

**Figure 1: Cumulative population as a function of PM2.5 (µg/m3) concentrations estimated**
a.Algeria, b.Burkina Faso, c.Burundi, d.Cameroon, e.Ivory Cost, f.Guinea, g.Madagascar, h.Mali, i.Morocco, j.Democratique Republic of the Congo, k.Senegal, l.Tunisia
